# Propolis-Loaded Poly(lactic-*co*-glycolic Acid) Nanofibers: An *In Vitro* Study

**DOI:** 10.1021/acsomega.3c09492

**Published:** 2024-03-15

**Authors:** Fulya Geyik, Seçil Kaya, Duygu Elif Yılmaz, Hasan Demirci, İlkgül Akmayan, Tülin Özbek, Serap Acar

**Affiliations:** † Faculty of Chemical and Metallurgical Engineering, Department of Bioengineering, Yildiz Technical University, Istanbul 34220, Turkey; ‡ Department of Nephrology and Medical Intensive Care, Charité-Universitätsmedizin Berlin, Berlin 10117, Germany; § Institute of Functional Anatomy, Charité-Universitätsmedizin Berlin, Berlin 10115, Germany; ∥ Faculty of Arts and Sciences, Department of Molecular Biology and Genetics, Yildiz Technical University, Istanbul 34220, Turkey

## Abstract

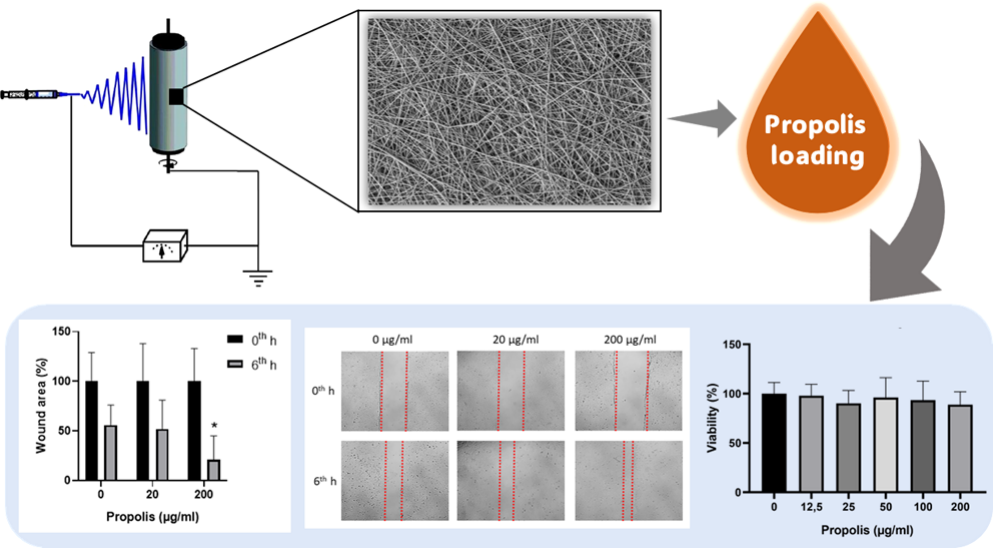

Nanofibers have high potential through their high porosity, small pore sizes, lightweight materials, and their ability to mimic the extracellular matrix structure for use in the manufacture of wound dressings for wound treatment. In this study, poly(lactic-*co*-glycolic acid) (PLGA) nanofibers were produced by electrospinning. Propolis was loaded into the PLGA nanofibers by the dropping method. The average diameters and effects of propolis loading on the morphology of 37.5, 50, and 100% propolis-loaded PLGA nanofibers (PLGA-P37.5, PLGA-P50, and PLGA-P100) were evaluated by scanning electron microscopy (SEM). The successful loading of propolis into PLGA nanofibers was confirmed with Fourier transform infrared spectroscopy (FTIR) analysis. *In vitro* propolis release was examined at physiological pH. The antioxidant activity of propolis-loaded nanofibers was studied with 2,2-diphenyl-1-picrylhydrazyl (DPPH). Antimicrobial activities of the nanofibers against *Escherichia coli*, *Staphylococcus aureus* and *Candida albicans* strains were determined by the disk diffusion method. Consequently, PLGA-P50 and PLGA-P100 showed high antimicrobial activity on *S. aureus* and *C. albicans*. Cell viability was tested by 3-[4,5-dimethylthiazole-2-yl]-2,5-diphenyltetrazolium bromide (MTT) assay, and propolis-loaded PLGA nanofibers were found to be biocompatible with human fibroblast cells. In the wound scratch assay, propolis-loaded nanofibers supported wound closure with cell migration and proliferation. Thus, *in vitro* wound closure properties of propolis-loaded PLGA nanofibers were evaluated for the first time in the literature.

## 1 Introduction

Wound dressings are biomaterials that can protect the wound from infection-causing microorganisms and accelerate wound healing.^1^ Electrospinning is one of the techniques used in the production of wound dressing materials consisting of nanothick fibers. Electrospun dressings have advantages in terms of biocompatibility and wound healing with their ability to mimic the natural extracellular matrix structure.^2^ The electrospinning method offers a wide range of production possibilities using various natural and synthetic polymers. Generally, the natural polymers are collagen, gelatin, chitosan, and silk fibroin, while the synthetic polymers are polylactide (PLA), poly(lactic-*co*-glycolic acid) (PLGA), polycaprolactone (PCL), poly(vinyl alcohol) (PVA), and polyurethane (PU). These polymers are extensive materials for the production of nanofibers because of their excellent properties.^3^ PLGA is one of the most widely used nanosystems developed for biomedical applications and synthesized by polycondensation of monomer units of poly(lactic acid) (PLA) and poly(glycolic acid) (PGA) polymers, especially by ring-opening copolymerization for high-molecular-weight copolymer production. PLGA is a promising material because of its biocompatibility, biodegradability, and versatility of properties. The most important advantages of PLGA are its monomer composition, molecular weight, crystallinity, and ability to produce polymers with different physicochemical properties by changing their properties. Other advantages of PLGA are that it is a polymer approved by the Food and Drug Administration (FDA), it is versatile in performance, biocompatible, biodegradable, and does not show toxic and immunogenic effects.^4^

Propolis is a natural substance produced by bees using plant resins and their enzymes to protect their hives.^5,6^ This product, commonly referred to as a “natural antibiotic”, has an important role in protecting, reinforcing, and repairing the hives. It is also a barrier to molds and yeasts, bacteria, and viruses.^7^ The studies have been performed on more than 300 compounds present in the structure of propolis. The composition of propolis depends on climatic conditions, time of collection, collector type, water and food availability, and other environmental factors.^7,8^ Generally, the most important compounds in propolis are flavonoids, phenylpropanoids, cinnamic acids and their esters, glycerides, and caffeic acid phenethyl ester.^9^ Also, studies have shown that propolis with these compounds has some important biological activities such as antibacterial,^10^ antifungal,^11^ antiviral,^12^ anti-inflammatory, antioxidant, anticancer, and immunomodulatory.^13^ Propolis as a traditional substance is used for skin regeneration and wound healing.^14^ In our study, we introduced a novel active loading method by loading propolis into an electrospun PLGA membrane using the dropping method, marking the first instance in the literature. Additionally, for the first time, we investigated the wound scratch assay of propolis-loaded PLGA nanofibers.

Propolis-loaded PLGA nanofibers have shown significant tissue repair in a domestic pig with contact burn.^15^ Stojko et al.^16^ have declared that propolis-PLGA nonwoven dressings have a healing effect on a domestic pig with third-degree burns. Alberti et al.^17^ have produced a wound dressing material with propolis nanoparticles formulation and electrospun PVA. They have found the wound closure percentage of this material to be 68.8% *in vivo*. *In vitro* wound scratch assay has been done before for propolis extracts and it has been concluded that propolis accelerates wound healing.^18^ However, there is no *in vitro* wound healing study of propolis-loaded electrospun PLGA membranes. In the literature, propolis has been loaded into nanofibers by the blending method. In this method, the active substance and polymer are blended in the same solvent. Therefore, it is a challenging method for substances that are poorly soluble in the solvent of the polymer. Additionally, the membrane obtained by using the electrospinning technique is not entirely utilizable. The edge parts of the membrane tend to be thinner, and an indeterminate portion of the fibers in electrospinning is collected outside the collector.^19^ In the present study, propolis has been loaded into PLGA nanofibers by the dropping method.^20^ In the dropping method, the drug solution is prepared and dropped into the electrospun membrane using an automatic pipette. The dropping method is a modified version of the dipping method, which is one of the active loading methods in the literature.^21,22^ In the dropping method, the required amount of solution is prepared, and the substance can be loaded onto a membrane of a suitable size. This enables the reduction of waste formation, consequently minimizing the environmental impact. Additionally, it is believed to address the challenge of electrospinning that arises from the nonsolubility of the substance and polymer in the same solvent.^20^

This study focuses on propolis-loaded electrospun PLGA membrane production and characterization. PLGA membrane was fabricated by the electrospinning method. A commercially available water extract of propolis was loaded into the membrane by a dropping method. In this work, the loading of propolis into electrospun membranes by the dropping method and wound scratch analysis of propolis-loaded PLGA nanofibers were reported for the first time. The nanofibers were characterized by scanning electron microscopy (SEM) and Fourier transform infrared spectroscopy (FTIR) analyses. Propolis release from PLGA nanofibers was monitored with absorbance measurement in a UV–vis spectrophotometer. The antioxidant activity of propolis-loaded PLGA nanofibers was investigated by the DPPH method. Their antimicrobial effects on *Staphylococcus aureus*, *Escherichia coli*, and *Candida albicans* were tested by the disk diffusion method. And *in vitro* cell viability and wound scratch assays were studied on human fibroblast cell lines.

## 2 Experimental Section

### 2.1 Materials

Poly(lactic-*co*-glycolic) acid (PLGA, *M*_w_ = 76–115 kDa, lactide/glycolide = 75:25), dimethylformamide (DMF), dichloromethane (DCM), penicillin–streptomycin, DPPH, and ethanol (EtOH) were purchased from Sigma-Aldrich (Missouri, USA). Dulbecco’s modified Eagle’s medium (DMEM) was obtained from PAN-Biotech (Bavaria, Germany). Fetal calf serum (FCS), Mueller Hinton agar (MHA), and Sabouraud dextrose agar (SDA) were obtained from Thermo Fisher Scientific (Massachusetts, USA). 3-(4,5-Dimethylthiazol-2-yl)-2,5-diphenyltetrazolium bromide (MTT) was obtained from BioFroxx GmbH (Einhausen, Germany). BEE’O propolis water-soluble extract (0.15 g/mL propolis) was obtained from SBS Scientific Bio Solutions, Inc. Co. (Istanbul, Turkey).

### 2.2 Preparation of Propolis-Loaded PLGA Nanofibers by Electrospinning

27% w/v PLGA was dissolved into a DCM and DMF solvent mixture (DCM/DMF volume ratio of 1:2) by a magnetic stirrer and was obtained as PLGA solution. This solution was loaded into a 5 mL syringe, and voltage was applied at 15 kV (NE300, Inovenso, Inc., Turkey). The solution was then electrospun onto a cylindrical collector with a 200 rpm rotation speed with a pumping rate of 0.5 mL/h, and the needle tip-to-collector distance was fixed at 150 mm (room temperature and 75% humidity). The dropping method was used for propolis loading.^19^ Three propolis extract solutions were prepared with ethanol at 100, 50, and 37.5%. 100 μL of solutions was dropped into 1 × 1 cm^2^ PLGA membranes using an automatic pipette and dried at room temperature overnight. Thus, PLGA-P37.5, PLGA-P50, and PLGA-P100 were obtained. Since the extract used contained 0.15 g/mL propolis, the amounts of propolis in the solutions loaded on PLGA-P100, PLGA-P50, and PLGA-P37.5 membranes were 15, 7.5, and 5.625 mg, respectively.

### 2.3 Scanning Electron Microscopy (SEM)

SEM analysis was performed to determine the surface morphology and diameters of the nanofiber.^23^ Produced PLGA nanofibers with and without propolis were placed onto black carbon tape with a double side. The samples were then coated with a Au–Pd layer under vacuum to form the conductive layer required for the measurement and analyzed with SEM (Zeiss EVOLS10, Oberkochen, Germany). The average diameter (AD) and standard deviation (SD) were calculated by measuring the diameter of 30 randomly selected fibers with ImageJ software.^24^

### 2.4 Fourier Transforms Infrared (FTIR) Spectrometry

The analysis was performed on an FTIR spectrophotometer (Shimadzu, Kyoto, Japan) to confirm that the propolis used as an active substance was successfully loaded into the PLGA nanofibers. The FTIR analysis of the propolis-loaded nanofibers was carried out comparatively with the PLGA nanofibers and propolis. The infrared spectra of these samples were obtained in the range of 4000–600 cm^–1^ with 16 scans per sample and 4 cm^–1^ resolution.^25−27^

### 2.5 Loading Efficiency

The loading efficiencies of propolis-loaded PLGA nanofibers were determined using UV–vis spectrophotometry. 1 mL of ethanol was added to the propolis-loaded PLGA nanofibers. Samples, thoroughly mixed, were subjected to UV–vis absorbance measurements, and their concentrations were calculated using a pre-established calibration curve (*y* = 17.24*x*, *R*^2^ = 0.9998). The loading efficiencies were calculated using eq 1 based on the initially loaded amounts of propolis.^28^ This experiment was repeated three times, and average values and standard deviations (±SD) were calculated.
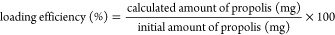
1

### 2.6 *In Vitro* Propolis Release

This study was performed using the Hall Barrientos et al. method.^29^ To investigate the release profile of propolis from the nanofibers, propolis-loaded PLGA membranes were cut to 1 cm^2^. These samples were incubated at 37 °C in a shaking incubator with 1.5 mL of phosphate-buffered saline (PBS) solution having a biological system pH (7.4). The liquid solution was then collected from the release medium for certain time intervals of 1st, 2nd, 3rd, 4th, 5th, 6th, 7th, 8th, 24th, 48th, 72nd, 96th, 120th, 144th, 168th, 192nd, and 264th hour, respectively, and fresh PBS was added to the release medium to continue the release process. The absorbance values were measured with a UV–vis spectrophotometer (Shimadzu, Kyoto, Japan) at 323 nm, and propolis concentrations were determined using a standard calibration curve (*y* = 5569.8*x*, *R*^2^ = 0.9957).^30^ The cumulative release (%) was calculated according to eq 2. When the release was calculated, the amount of propolis loaded on each membrane and the loading efficiency were considered. The release study was carried out three times, and ±SD and average values were calculated.
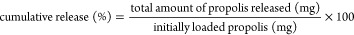
2

### 2.7 Antioxidant Activity

The antioxidant activity of propolis-loaded PLGA nanofibers was studied by the DPPH method as proposed by Nithya and Madhavi.^31^ PLGA nanofibers without propolis (PLGA), 37.5% propolis-loaded (PLGA-P37.5), 50% propolis-loaded (PLGA-P50), and 100% propolis-loaded (PLGA-P100) nanofibers were put into the 1.5 mL of DPPH solutions (100 μM) and kept in a dark medium for 30 min. Then, their spectroscopic measurements were performed at 517 nm and antioxidant activities were calculated according to eq 3(32)
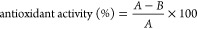
3

Where *A* and *B* indicate the absorbance values of the DPPH solution and sample-treated DPPH solution, respectively.

### 2.8 Antimicrobial Activity

The disk diffusion method was used to determine the antimicrobial activity of propolis-loaded PLGA nanofibers according to EUCAST standards.^33^ In this study, we compared *E. coli*, *S. aureus*, and *C. albicans* strains because they are dominant pathogens in wound infections.^34^ 1.5 to 3 × 10^8^ CFU/mL microbial suspensions were swabbed on the Mueller Hinton agar (MHA) plate. Sabouraud dextrose agar (SDA) was used for *C. albicans*. 1 cm^2^ of PLGA, PLGA-P37.5, PLGA-P50, and PLGA-P100 were placed on the surface of the agar. After 24 h of incubation at 37 °C, the inhibition zones of nanofibers on microorganisms were evaluated.^35^

### 2.9 Cell Culture

Human fibroblast cells were grown in 75 cm^2^ flasks in DMEM including 10% fetal calf serum and 1% penicillin–streptomycin in a humidified incubator at 37 °C with 5% CO_2_ (Binder GmbH).

### 2.10 *In Vitro* Viability

Cell viability was evaluated by MTT assay.^36^ Approximately 10^4^ cells were inoculated in each well of a 96-well plate and incubated at 37 °C for 24 h. In the meantime, a propolis-loaded PLGA membrane was incubated in 5 mL of DMEM at 37 °C, shaking for 24 h to release the drug. Subsequently, released propolis was diluted to the following concentrations: 12.5, 25, 50, 100, and 200 μg/mL, and an appropriate amount of propolis-containing media was added to 100% confluent cells. The treatment was carried out for 24 h, and the control group was treated with a medium without any drug in parallel. To prepare the MTT solution, 5 mg/mL MTT was dissolved in phosphate-buffered saline (PBS) and sterilized by filtration (0.22 μm, Whatman, U.K.). After the treatment, 10 μL of MTT solution was pipetted into each well and incubated at 37 °C for 2 h. Afterward, the medium was aspirated, and precipitated formazan crystals in each well were dissolved in 100 μL of dimethyl sulfoxide (DMSO) and incubated gently shaking at 37 °C for 15 min. The light absorbance was measured at 560 nm with a microplate reader (Biochrom Asys Expert 96, U.K.).^37,38^ The viability study was carried out three times, and ±SD and average values were calculated. The growth in drug-free control was considered to be 100% viable, and relative viability was calculated according to eq 4
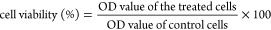
4

### 2.11 *In Vitro* Wound Scratch Assay

*In vitro* wound scratch assay was conducted in 24-well plates including 14 mm coverslips.^39^ A suspension of 5 × 10^4^ human fibroblast cells was inoculated into each well and incubated at 37 °C until the cell monolayer covered the surface of the coverslips entirely. Subsequently, the medium was changed with 20 or 200 μg/mL propolis-containing media and a longitudinal line was scratched with a sterile 200 μL pipette tip across the coverslip. The control group was treated with only a medium without any drug. The imaging was enabled with a light microscope (Leica, Wetzlar, Germany) with 40× magnification in the zeroth and sixth hours. The wound area was measured by using ImageJ software (NIH).

### 2.12 Statistical Analysis

In the statistical analysis, ±SD values were calculated and *p* < 0.05 was considered as a significance level.^40^

## 3 Results and Discussion

### 3.1 Scanning Electron Microscopy (SEM)

**Figure 1:**
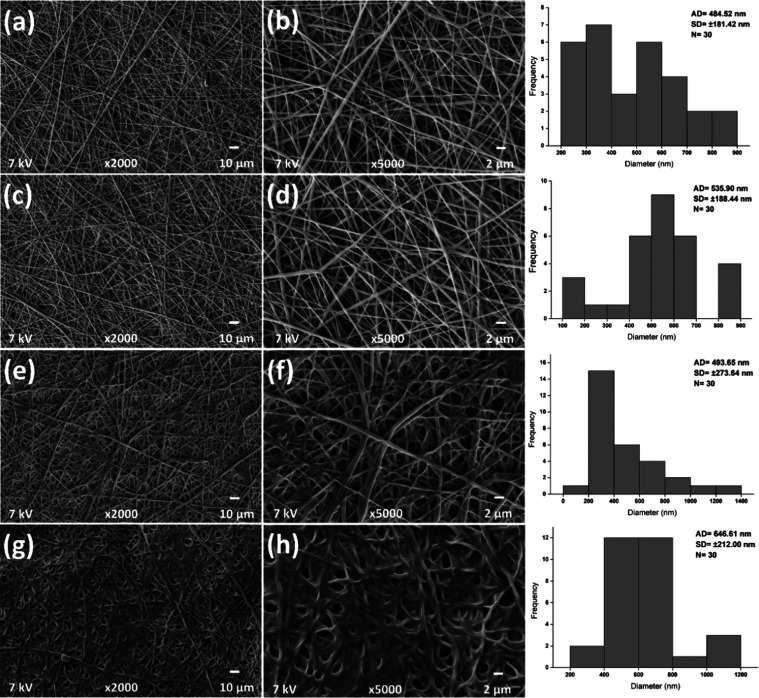
SEM images of PLGA (a, b), PLGA-P37.5 (c, d), PLGA-P50 (e, f), and PLGA-P100 (g, h) and their size distribution histograms.

In this study, PLGA nanofibers were produced by the electrospinning method, and propolis was loaded onto the nanofibers. The average fiber diameters of PLGA, PLGA-P37.5, PLGA-P50, and PLGA-P100 were measured as 484.52 ± 181.42, 535.90 ± 188.44, 493.65 ± 273.64, and 646.61 ± 212.00 nm, respectively (Figure 1). The average diameter of PLGA-P100 is significantly larger than propolis-free nanofibers (*p* < 0.05) (Figure 1g,h). Propolis loading has increased the diameter and standard deviation of the PLGA nanofibers. Similar results have been obtained by Asawahame et al.;^13^ it was observed that the diameters of nanofibers increased with the effect of propolis. It has been indicated that the reason for this increase may be the effect of the propulsive forces and high viscosity of the components in the propolis extract added to the structure.^13^ But, in the present study, propolis has been loaded into PLGA nanofibers by the dropping method. Therefore, the increase in the diameter of the nanofibers can be attributed to their swelling by absorbing propolis extract. It has been observed in the literature^41^ that nanosized fibers tend to better support cell viability compared to those in the microscale. In this regard, it can be stated that the fiber size obtained in our study is suitable for biocompatibility.

### 3.2 Fourier Transform Infrared (FTIR) Spectroscopy Analysis

**Figure 2:**
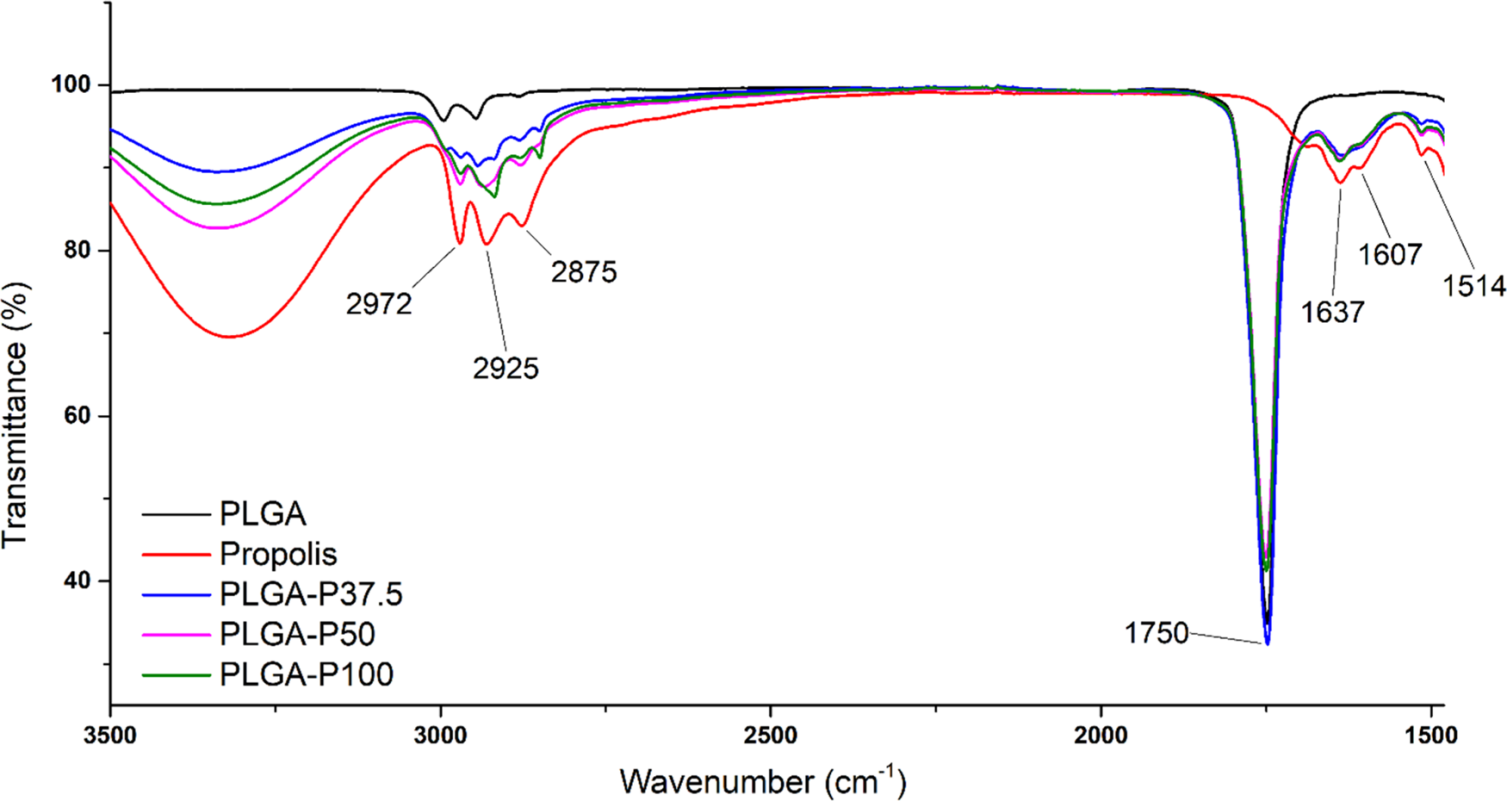
FTIR spectra of PLGA, propolis, PLGA-P37.5, PLGA-P50, and PLGA-P100.

FTIR analysis was investigated to determine the functional groups of samples and the effect of propolis loading in the nanofibers. FTIR spectra of propolis, PLGA, PLGA-P37.5, PLGA-P50, and PLGA-P100 are shown in Figure 2. The band of C=O groups (1750 cm^–1^) in the structure of PLGA is seen in the spectra of PLGA, PLGA-P37.5, PLGA-P50, and PLGA-P100. 1637, 1607, and 1514 cm^–1^ bands indicate C=C groups in phenolic compounds of propolis structure (caffeic acid, cinnamic acid, ferulic acid, etc.).^42^ The presence of these bands in the propolis-loaded nanofibers’ spectra indicates propolis’s successful loading. And, 2972, 2925, and 2875 cm^–1^ bands can be attributed to C–H stretching.

### 3.3 Loading Efficiency

The loading efficiencies of PLGA-P100, PLGA-P50, and PLGA-P37.5 were found to be 87.38 ± 12.86, 85.94 ± 7.6, and 88.35 ± 6.08%, respectively. It was determined that loading different amounts of propolis did not make a significant difference in the loading efficiency (*p* > 0.05). The high standard deviation of PLGA-P100 may be attributed to the partially challenging loading of propolis extract owing to its oil-like structure. Thus, it is observed that the dilution of the propolis extract with ethanol leads to a more uniform loading efficiency. Previously, Shakiba et al.^43^ have discovered that the loading efficiency of nanofibers produced using curcumin-loaded halloysite nanotubes by blending method is approximately 39%. In our study, the high loading efficiency may be associated with the use of the dropping method. High loading efficiency may confer an advantage in terms of enhanced utilization of the active substance in wound dressings.

### 3.4 *In Vitro* Propolis Release

**Figure 3:**
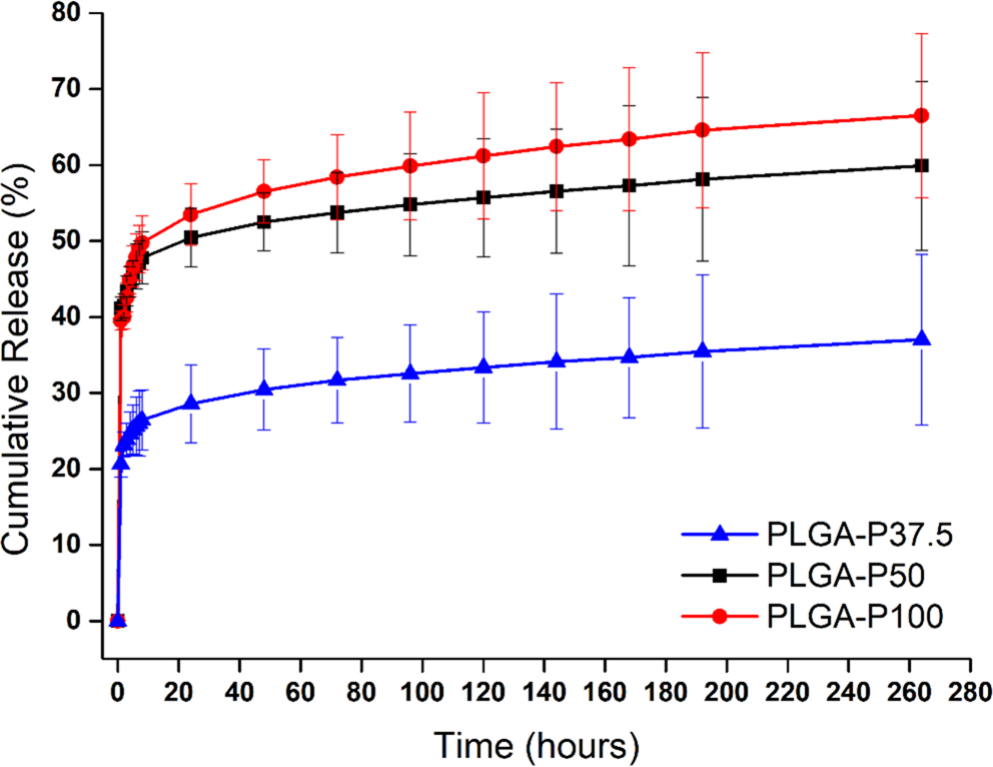
Cumulative propolis release results of PLGA-P37.5, PLGA-P50, and PLGA-P100.

Propolis at three concentration values (37.5, 50, and 100%) were loaded onto the membranes and placed in the release medium. The absorption values were measured by a UV–vis spectrophotometer to determine the concentrations of samples taken from the release medium at regular intervals. The concentration values corresponding to these absorption values were calculated by using the calibration chart prepared previously. With the help of concentration values, cumulative release percentages were calculated, and time-dependent graphs of release percentages were obtained. The cumulative release percentages of propolis-loaded nanofibers at different concentrations over time are given in Figure 3. The initial release rates of PLGA-P37.5, PLGA-P50, and PLGA-P100 at the end of 264 h are 37.02 ± 11.2, 59.88 ± 11.12, and 66.51 ± 10.8%, respectively. The statistical results have shown that there is no significant difference between PLGA-P50 and PLGA-P100 regarding the propolis release. The release values of PLGA-P37.5 are significantly lower than those of PLGA-P50 and PLGA-P100. In the study of Zeighampour et al.,^44^ propolis was released from PVA nanofibers at a rate of 80–85% after 100 h. The fact that the release rate from PVA nanofibers is higher than that of PLGA nanofibers can be attributed to the hydrophilic nature of PVA. In our study, a slower release was observed due to the hydrophobic nature of PLGA. Additionally, based on the release results, it can be stated that propolis effectively penetrated PLGA nanofibers, and the phenolic compounds within it, such as caffeic acid phenethyl ester,^45^ likely underwent physical interactions with PLGA. Thus, PLGA-P50 and PLGA-P100 may be potential wound dressing materials for wounds with delayed healing because of their long-time propolis release.

### 3.5 Antioxidant Activity

The antioxidant activity of propolis-loaded PLGA nanofibers was tested by the DPPH radical scavenging assay. Antioxidant activity percentages of PLGA, propolis, PLGA-P37.5, PLGA-P50, and P100 are 5.7 ± 0.11, 68.6 ± 0.84, 78.1 ± 1.58, 85.3 ± 0.34, and 88.4 ± 0.52, respectively. Propolis loading significantly increased the radical scavenging performance of PLGA nanofibers. The distribution of propolis in the DPPH solution was regulated by nanofibers, and thus, higher activity was observed in propolis-loaded PLGA nanofibers. It is seen that the antioxidant activity increases as the amount of propolis increases. Propolis-impregnated cellulose acetate/polycaprolactone nanofibers showed 60–80% radical scavenging activity. Differently, free propolis has higher activity than that loaded into nanofibers.^46^ In our study, it can be said that loading nanofibers improves the antioxidant activity of propolis. The use of propolis with antioxidant properties may improve oxidative-stress-related delayed wound healing. The ability of propolis to scavenge free radicals is well known in the literature. It has been indicated that propolis obtained from various regions may exhibit different antioxidant activities.^47^ In our study, the free radical scavenging capacity of the propolis extract loaded onto PLGA nanofibers is significantly high. Propolis-loaded PLGA nanofibers can provide significant antioxidant activity for healing wounds and burn.

### 3.6 Antimicrobial Activity

**Figure 4:**
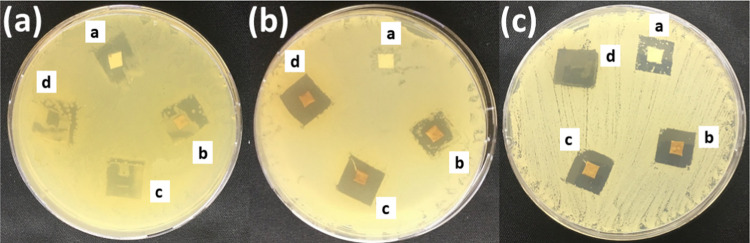
Inhibition zones of (a) PLGA, (b) PLGA-P37.5, (c) PLGA-P50, and (d) PLGA-P100 on (a) *E. coli*, (b) *S. aureus*, and (c) *C. albicans* strains.

Disk diffusion method was used to determine the antibacterial and antifungal effect of PLGA nanofibers loaded with different concentrations of propolis samples against *E. coli*, *S. aureus*, and *C. albicans* strains (Figure 4 and Table 1). PLGA-P50 and PLGA-P100 showed very low inhibition effects on *E. coli*. PLGA-P37.5 has no inhibition zone against *E. coli*. Similarly, zein/propolis nanofibers have antimicrobial activity only on Gram-positive strains and fungi.^48^ The lower activity of *E. coli* as a Gram-negative bacteria may be due to it having a complex outer membrane in addition to the peptidoglycan layer.^49^ PLGA nanofibers without propolis do not show an inhibition zone alone, whereas PLGA-P50 and PLGA-P100 have high antimicrobial activity on *S. aureus* and *C. albicans*. PLGA-P50 and PLGA-100 have significantly higher activity than PLGA-P37.5 on bacteria strains. However, it can be said that the dose of propolis does not affect the antifungal activity.

**Table 1:** Inhibition Zone Diameters of Samples on Microbial Strains

sample	*E. coli* (mm)	*S. aureus* (mm)	*C. albicans* (mm)
PLGA			
PLGA-P37.5		9	14
PLGA-P50	5	15	15
PLGA-P100	5	14	15

*S. aureus* is the most well-known pathogen responsible for infections of diabetic wounds. *S. aureus* exacerbates infection in diabetic foot ulcers, delaying the healing process.^50^ The propolis-loaded PLGA nanofibers produced in our study have the potential to serve as a candidate wound dressing for the prevention of infections caused by *S. aureus*. *C. albicans* is known as a pathogen that can cause serious infections, particularly in thermal wounds. Developing an effective wound dressing against *C. albicans* is crucial for the treatment of life-threatening infections in burn patients.^51^ Our results indicated that nanofibers containing 50 and 100% propolis can be effective when used as potential wound dressings in *S. aureus*- and *C. albicans*-infected wounds.

### 3.7 Cell Viability

**Figure 5:**
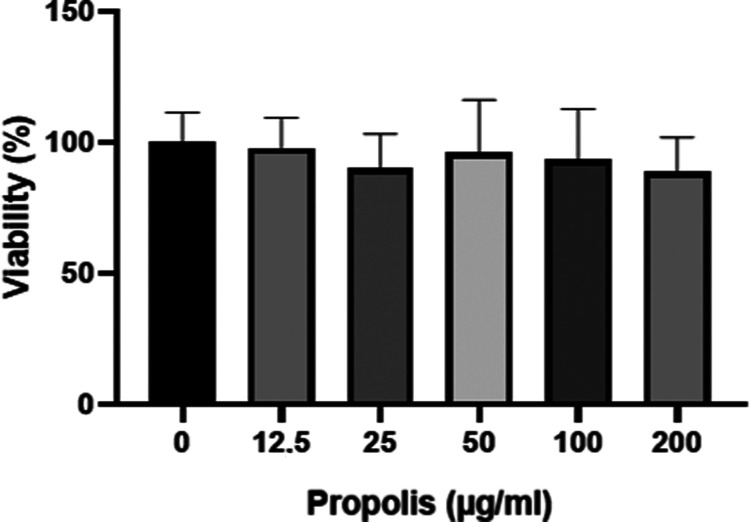
MTT assay results of propolis-loaded PLGA nanofibers.

PLGA-P50 was chosen for the cell viability and wound healing assays. Cytotoxicity of propolis-loaded PLGA was detected via the MTT assay. The results revealed that applied doses of propolis were not found to be cytotoxic. Even after the highest dose, which is 200 μg/mL, cellular viability was found to be 88.79 ± 17.65% within 24 h of the period (Figure 5). The release graph shows that propolis is released at a high rate in 24 h (Figure 3). We concluded that the propolis dose has no significant effect on biocompatibility (*p* > 0.05). The lack of a significant impact on cell viability with an increase in propolis dosage suggests that propolis can be used in a wide concentration range in nanofiber wound dressings. Electrospun nanofibers provide a suitable environment for cell adhesion and proliferation.^52^ And PLGA is a biocompatible polymer that is frequently used in drug delivery systems.^53^ Propolis-loaded nanofibers have also been found compatible with different cell lines.^54^ The biocompatibility of wound dressings is very important in terms of not creating an immune response in the wound area and not damaging healthy cells.^55^ Propolis-loaded PLGA nanofibers have the potential to be used as a wound dressing material due to their biocompatibility with fibroblast cells.

### 3.8 *In Vitro* Wound Scratch Assay

**Figure 6:**
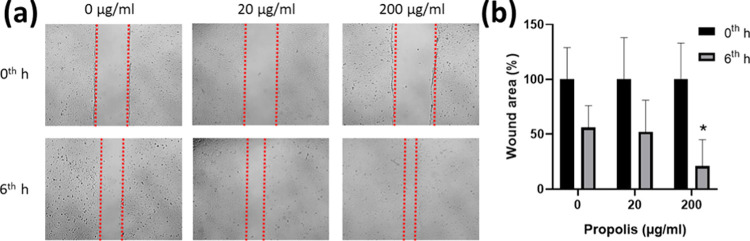
(a) *In vitro* wound scratch assay images and (b) wound area results of propolis-loaded PLGA nanofibers.

The *in vitro* wound healing properties of propolis-loaded PLGA nanofibers were evaluated by measuring the closure of the scratch area. After 6 h, the wound areas for 0, 20, and 200 μg/mL propolis were 56.1, 52.2, and 21.24%, respectively (Figure 6). The results demonstrated that cell migration and proliferation were significantly improved with 200 μg/mL propolis treatment compared to control after 6 h (*p* < 0.05). De Figueiredo et al.^56^ reported that PCL films with a release of ∼25 μg/mL propolis after 24 h had no significant effect on wound closure compared to the control. The effect of PCL films with higher propolis amounts on wound closure has been not examined.^56^ The results of our study have shown that 200 μg/mL propolis in the PLGA nanofibers supports wound healing; at the same time, it has no toxic effect on fibroblast cells. The wound healing properties of propolis-loaded nanofibers have been studied in a few *in vivo* studies. Shie Karizmeh et al.^57^ have produced a bilayer membrane with PCL/chitosan nanofibers on polyurethane (PU)-propolis foam for an *in vivo* study. The bilayer membrane treated wounds healed completely, while the PU-propolis foam provided a lower wound closure. In addition, the bilayer membrane group has exhibited effective wound healing, characterized by the formation of hair follicles and enhanced dermis.^57^ It can be concluded that electrospun membranes not only release propolis but also contribute to wound healing with their high surface area and porous structure. And, in burn treatment studies, propolis-loaded electrospun PLGA films have contributed to the restructuring of the epidermis and reduced the wound area in domestic pigs.^15,16^

In our study, propolis-loaded PLGA nanofibers increased the migration and proliferation of fibroblast cells and provided a high rate of wound closure. This study revealed that PLGA nanofibers loaded with propolis by the dropping method can be suitable materials for wound healing.

## 4 Conclusions

This study presents a different loading method and produces a propolis-loaded electrospun PLGA membrane for wound healing. It was observed that propolis loading affected the morphology of the nanofibers in the SEM images. PLGA nanofibers with a bead-free structure and a suitable fiber diameter (480–650 nm) in terms of biocompatibility have been manufactured by electrospinning. It was proved by FTIR analysis that propolis was attached to the surface of the nanofibers. Additionally, the dropping method resulted in a notably high loading efficiency (85–88%) of propolis. According to cumulative release study analysis, propolis was released from nanofibers for 264 h. As a drug delivery system, PLGA nanofibers can sustain the release of propolis to the wound site. The 88.4 ± 0.52% antioxidant activity of PLGA-P100 indicates that it could be an effective material in addressing delayed wound healing associated with a significant increase in the number of free radicals. A material has been produced that facilitates wound closure by promoting cell migration and proliferation. The nanofibers have a strong wound closure activity in the scratch assay (21.24% wound area for 200 μg/mL of propolis). It is a biocompatible material with fibroblast cells (88.79 ± 17.65% in 24 h for 200 μg/mL propolis); in this respect, it can be said to be a good tissue scaffold. Propolis-loaded PLGA nanofibers inhibited *S. aureus*, a Gram-positive bacteria, but did not show any inhibition zone against *E. coli*, a Gram-negative bacteria. Propolis-loaded nanofibers can be biomaterial that can support wound healing by preventing infection in wounds infected by Gram-positives and fungus. This comprehensive approach to wound healing, encompassing both drug delivery and antibacterial properties, positions propolis-loaded PLGA nanofibers as a valuable biomaterial for enhancing wound care outcomes.

## Data Availability

All data relevant to the study are included in the article.
